# Supplementary material for: Caenorhabditis diversity on Pohnpei, Micronesia, provides evidence that the Elegans Supergroup has its roots in the Americas and diversified in the Pacific en route to Asia
Source: bioRxiv. 2025 Sep 24:2025.09.22.677770. Preprint. [Version 1] doi: 10.1101/2025.09.22.677770 (PMC12485960; doi:10.1101/2025.09.22.677770)
Supplement: Supplement 3 [file NIHPP2025.09.22.677770v1-supplement-3.pdf]

bioRxiv preprint doi: https://doi.org/10.1101/2025.09.22.677770; this version posted September 24, 2025. The copyright holder for this preprint (which was not certified by peer review) is the author/funder, who has granted bioRxiv a license to display the preprint in perpetuity. It is made available under aCC-BY 4.0 International license.

DATA AVAILABILITY STATEMENT

Sample collection data are reported in Supplementary Tables. Type cultures of each new species are deposited at the *Caenorhabditis* Genetics Center, and cultures of all isolates are available from the authors. Ribosomal DNA sequences have been deposited in Genbank with identifiers PP955316-PP955322. RNAseq data and transcriptome assemblies are deposited with NCBI under BioProject ID PRJNA1128046.

SUPPLEMENTARY MATERIAL

Appendix: Descriptions of five new species

Supplementary Tables

Table S1. Transcriptomic Data. For each species, the table lists the BUSCO annotation of the transcriptome (percent complete, single copy, duplicate, fragmented, and missing), the identity of the sequence reference strain, the legacy species number for species that are or were previously known by temporary species numbers, the source of the sequence data (*e.g., Caenorhabditis* Genome Project CGP), the reference for the data, the nature of the data (transcriptome or genome sequence), and the file name for the source data.

Table S2. Biogeographic Data. For each species, the table lists the legacy species number, the species name, its known geographic distribution, and a citation for the distribution information. The remaining columns record 1 or 0 for the presence or absence of each species from the specified geographic region, as shown in Figures 6 and 7, and indicate whether the species is included in the biogeographic analysis. Species were excluded if they were globally distributed, if their distributions were ambiguous (*e.g., C. sp. 2*), or if they fall outside the clade defined by the Elegans and Drosophilae Supergroups.

Table S3. Survey Results. This table provides sample-level information for each of the substrates examined as part of our survey of the island. It includes the 400 rotting samples at the core of the survey, and an additional 14 non-rotting samples including fresh figs and live invertebrates. The columns list, for each sample, the sample ID (corresponding to the barcoded sample bag), the Collection Set (one for each locality-day), the C-plate summary (reporting at a high level what worms if any emerged from each sample's Baermann Funnel extraction onto a petri dish, the C-plate), the latitude, longitude, and elevation of the sample, the number of *Caenorhabditis* worms identified to species level, whether the sample included gonochoristic *Caenorhabditis* worms of a single sex only (precluding establishment of a culture), and then a series of columns reporting presence (1) or absence (0) of each species of *Caenorhabditis*. The remaining columns report the general substrate class, the substrate identity, whether the sample was rotting material (1 for yes, 0 for no), whether the sample was found on the ground or at an elevated position, the Pohnpei-specific vegetation type, the general landscape classification (following *Caenorhabditis* conventions), the substrate temperature, the ambient temperature, and the collection date.

Table S4. All *Caenorhabditis* Isolates. This table provides worm-isolate-level information for each of the individuals isolated from each of the 400 survey samples and 111 fig and leaf-litter samples from the spatial sampling arrays at Sokehs Ridge. For each isolate, the table lists the Isolate ID (Sample ID as in Table S3 with the addition of an upper-case letter for the Isolate ID), the species of *Caenorhabditis*, the QG number that represents the unique strain name for the cryopreserved culture established from the isofemale or isohermaphrodite isolate (following *C. elegans* genetics convention, QG indicates the laboratory and strains are assigned numbers sequentially), the Sample ID, a Yes or No as to whether the sample is part of the Sokehs Ridge spatial sample set, and then the Collection

bioRxiv preprint doi: https://doi.org/10.1101/2025.09.22.677770; this version posted September 24, 2025. The copyright holder for this preprint (which was not certified by peer review) is the author/funder, who has granted bioRxiv a license to display the preprint in perpetuity. It is made available under aCC-BY 4.0 International license.

Set, latitude, longitude, and elevation, as in Table S3. The remaining columns report the general substrate class, the substrate identity, whether the sample was rotting material (1 for yes, 0 for no), whether the sample was found on the ground or at an elevated position, the Pohnpei-specific vegetation type, and the general landscape classification.

Table S5. Mating Tests. This sheet contains four tables, each describing the results of experimental test crosses among closely related strains or species. The cross data allow us to establish *C. ileile, C. nansapw, C. mwetiwel,* and *C. pwilidak* as biological species incompatible with closely related species, and they show that Pohnpeian isolates QG4816 and QG5228 are compatible with known isolates of *C. zanzibari* and *C. parvicauda*, respectively.

Table S6. Spatial Sampling. This table provides sample-level information about the 111 figs and leaf-litter samples collected from the spatial arrays shown in Figure 5. One sample, the fig from quadrat 2, position B2, was lost and is not reported here. For each of the remaining samples, the table shows the Sample Name, the sample ID in C-plate format, the number of individuals identified as *C. pwilidak, C. briggsae,* or *C. nansapw*, and the total number of *Caenorhabditis* species found among the identified worms. The WormDensity column reports an ordered factor describing the overall number of worms recovered from the sample's Baermann Funnel, as follows: 0, 0 worms; 1, 1-5 worms; 2: 6-10 worms; 3: 11-50 worms; 4: 51-100 worms; 5: 101-500 worms; 6: 501-1000 worms. The remaining columns report the sample's quadrat (1, 2, or 3), row, column, and type (fig or leaf litter). The 9 figs sampled from the central position of each quadrat to represent a range of rottenness levels are designated by "R" as their value under "quad_column," and their rottenness level is indicated by the values 1-9 in the under "quad_row."

Table S7. Biogeographic Analyses. This sheet contains two tables describing statistical results from biogeographic models. The upper table lists the negative log likelihoods for each of six models, along with their parameter estimates. Models incorporating jump speciation (+J) confer much higher likelihoods than those without, and the DEC+J model confers the highest likelihood. The lower table tests the significance of incorporating the J parameter, in each case finding that it significantly improves the likelihood.

Supplementary Files

File S1. Statistics of patch colonization. This is a .R file containing scripts that estimate parameters of patch colonization and simulate populations to estimate aggregation statistics.

File S2. *Caenorhabditis* phylogeny. This is a Newick tree file containing the unrooted species tree estimated in Astral from 2955 gene trees. The branch lengths were then estimated in iqtree using the concatenated amino-acid sequences of 1,913 single-copy orthologs. This is the tree plotted in figure 6.

File S3. Ultrametric tree for biogeographic analysis. This is the input Phylogeny file for BioGeoBEARS analysis. It is a Newick tree file containing the rooted ultrametric species tree, as plotted in figure 7.

File S4. Biogeography file. This is the input Geography file for BioGeoBEARS analysis. The underlying data are sourced and adapted as described in Table S2.

ACKNOWLEDGEMENTS

We gratefully acknowledge the assistance of our guides and consultants in Pohnpei, Holden Pelep (Kitti municipality) and Zelnick Moses (U municipality). We are grateful for support and assistance from Roseo Marquez and Tamara Greenstone Alefaio (Micronesia Conservation Trust), Eugene Joseph (Conservation Society of Pohnpei), Hubert Yamada (Pohnpei State Resources and Development), Vanessa Fread and Dave Mathias (FSM Resources and Development), Eugene

bioRxiv preprint doi: https://doi.org/10.1101/2025.09.22.677770; this version posted September 24, 2025. The copyright holder for this preprint (which was not certified by peer review) is the author/funder, who has granted bioRxiv a license to display the preprint in perpetuity. It is made available under aCC-BY 4.0 International license.

Eperiam (Division of Natural Resource Management), Chief Minister Rufino Primo (U Municipal Government), and Chief Mihkel Bernardo, Ryan Agrippa, and Kenny Bernardo (U). Thanks to Micronesian Productions for the translation of the summary into Pohnpeian. We thank Derin Çaglar for help in the lab, NYU GenCore, supported by the Zegar Family Foundation, for sequencing support, and NYU HPC for computational support. We thank the *Caenorhabditis* Genetics Center (supported by NIH P40 OD10440) and the Fitch Lab at NYU for worm strains, WormBase and the *Caenorhabditis* Genomes Project for data, and the researchers who contributed strains and information to the CGP and to the worm evolution community wiki. Thanks to Manpreet Katari, Ryan Baugh, Zoltan Szuts, Erik Andersen, Justin Bernstein, and Jesse Czekanski-Moir for helpful advice. Apologies to Samuel Beckett for borrowing "birth astride of a grave" for island biogeography. The laboratory and computational parts of this work were supported by NIH R35GM141906.

bioRxiv preprint doi: https://doi.org/10.1101/2025.09.22.677770; this version posted September 24, 2025. The copyright holder for this preprint (which was not certified by peer review) is the author/funder, who has granted bioRxiv a license to display the preprint in perpetuity. It is made available under aCC-BY 4.0 International license.

Appendix:
Descriptions of five new species

Following the approach of Félix *et al.* (2014), and in recognition that many *Caenorhabditis* species are morphologically cryptic, we employ experimental crosses to diagnose species according to the Biological Species Concept. Photographs of male tails are provided in Figure A1 in accordance with the International Code of Zoological Nomenclature guidelines for species descriptions.

The electronic edition of this article conforms to the requirements of the amended International Code of Zoological Nomenclature and new names contained here are available under that Code from the electronic edition of this article. This published work and its nomenclatural acts are registered in ZooBank. The ZooBank LSIDs (Life Science Identifiers) can be resolved and the associated information viewed through any standard web browser by appending the LSID to the prefix "http://zoobank.org/". The LSID for this publication is: urn:lsid:zoobank.org:pub:F8CC909D-582B-4EAC-A46F-E326F00E6F01

Methods
Worms were cultured at 25°C on NGMA plates seeded with OP50-1 *E. coli* bacteria. Adult males, 1 day post L4, were heat-killed and mounted on 4% agar pads on microscope slides and imaged on a Zeiss AxioImager M2 microscope with DIC optics and EC Plan-Neofluar 100x/1.3 objective.

Table A1. Summary of new species

Temporary number	Species name	Type Strain	Three-letter abbreviation	ITS2 accession	Transcriptome accession
72	C. *losolos*	QG4708	Clo	PP955316.1	GKWV00000000.1
73	C. *mwetiwel*	QG4797	Cmw	PP955317.1	GKWX00000000.1
74	C. *nansapw*	QG4644	Cna	PP955318.1	GKWW00000000.1
75	C. *ileile*	QG4848	Cil	PP955319.1	GKWQ00000000.1
76	C. *pwilidak*	QG4628	Cpw	PP955320.1	GKWU00000000.1

Species Declarations

Caenorhabditis losolos* Rockman, Tintori, Nguyen, & Yomai *sp. nov.
ZooBank identifier urn:lsid:zoobank.org:act:86DCD029-9506-4ADA-A6BF-214A364F1EF2
= *Caenorhabditis sp. 72* (temporary number)
Type material: The type isolate by present designation is isofemale culture QG4708, cryopreserved as a living stock at the *Caenorhabditis* Genetics Center, Minneapolis, MN. Derived from a single female collected in Kitti, Pohnpei, Federated States of Micronesia (coordinates N 6.8652, E 158.173, elevation 167 m, 6 December 2023. The holotype is deposited at the *Caenorhabditis* Genetics Center.
Etymology: Losolos is a Pohnpeian word meaning lush, in reference to the type locality.
Diagnosis: The species is diagnosed and delineated by fertile crosses with type isolate QG4708 in both directions, yielding fertile male and female offspring that are interfertile with one another and with both parental isolates in both directions. The species differs in its ITS2 DNA sequence from all other named species, including those listed in Félix *et al.* 2014; Huang *et al.* 2014; Ferrari *et al.* 2017; Slos *et al.* 2017; Kanzaki *et al.* 2018; Crombie *et al.* 2019; Stevens *et al.* 2019; Dayi *et al.* 2021; Sloat *et al.* 2022; Devi *et al.* 2025; and all other species described in this paper. The phylogeny inferred from transcriptome sequences places *C. losolos* on a long branch with no close relatives, sister to a clade

bioRxiv preprint doi: https://doi.org/10.1101/2025.09.22.677770; this version posted September 24, 2025. The copyright holder for this preprint (which was not certified by peer review) is the author/funder, who has granted bioRxiv a license to display the preprint in perpetuity. It is made available under aCC-BY 4.0 International license.

that includes *C. japonica, C. imperialis*, and *C. spp. 25* and *49,* and new Pohnpeian species *C. mwetiwel, C. nansapw,* and *C ileile*. Experimental crosses with the other species described in this paper (including *C. mwetiwel, C. nansapw,* and *C ileile*) did not produce fertile progeny.
Type locality: The type isolate was recovered from a rotting *Citrus aurantifolia* collected from an agroforest plot at Pehleng, Kitti, on 6 December 2023. Overnight incubation in a Baermann funnel yielded approximately 20 worms, of which all but one were *Caenorhabditis* males and females. Four isofemale lines were established by picking single females on 7 December 2023 and transported to New York. These four isolates (QG4708, QG5173, QG5234, and QG5242) are all *C. losolos*. Eight additional isolates of this species were recovered from three other agroforest samples: a rotting nihn fig (*Ficus tinctoria*) in Kitti, and a rotting Hibiscus flower and a rotting cherry (*Muntingia calabura*) in U.
Morphology notes: The male tail has the typical characteristics of Elegans supergroup species, including a heart-shaped fan with a serrated edge and terminal notch (Figure A1). Rays 1, 5, and 7 open dorsally. Ray 3 is equally distant from rays 2 and 4. Ray 4 is shorter and skinnier than ray 5. Spicules are long and slender with pointy tips, and the precloacal lip has the characteristic hook shape.
Reproduction: This species has separate males and females. Mating is in the parallel position on plates and females are oviparous.

Caenorhabditis mwetiwel* Rockman, Tintori, Nguyen, & Yomai *sp. nov.
ZooBank identifier urn:lsid:zoobank.org:act:A4640623-3376-45F9-81BC-EE73C01135FF
= *Caenorhabditis sp. 73* (temporary number)
Type material: The type isolate by present designation is isofemale culture QG4797, cryopreserved as a living stock at the *Caenorhabditis* Genetics Center, Minneapolis, MN. Derived from a single female collected in Nett, Pohnpei, Federated States of Micronesia (coordinates N 6.9582, E 158.2096, elevation 34 m, 13 December 2023. The holotype is deposited at the *Caenorhabditis* Genetics Center.
Etymology: Mwetiwel is a Pohnpeian word meaning garden, a reference to the type locality.
Diagnosis: This species reproduces with separate males and females. The species is diagnosed and delineated by fertile crosses with type isolate QG4797 in both directions, yielding fertile male and female offspring that are interfertile with one another and with both parental isolates in both directions. The species differs in its ITS2 DNA sequence from all other named species, including those listed in Félix *et al.* 2014; Huang *et al.* 2014; Ferrari *et al.* 2017; Slos *et al.* 2017; Kanzaki *et al.* 2018; Crombie *et al.* 2019; Stevens *et al.* 2019; Dayi *et al.* 2021; Sloat *et al.* 2022; Devi *et al.* 2025; and all other species described in this paper. The phylogeny inferred from transcriptome sequences places *C. mwetiwel* as sister to a clade of *C. imperialis, C. nansapw,* and *C ileile.* Reciprocal experimental crosses with these species did not produce fertile hybrids, though all crosses but one produced viable, infertile F_1 adults (Table S5). Crosses between *C. mwetiwel* males and *C. imperialis* females resulted in dead embryos only. The clade that includes these species and *C. mwetiwel* is in turn sister to the clade of *C. sp. 25* and *C. sp. 49.* Reciprocal experimental crosses with these species produced dead embryos, plus a small number of arrested L1 larvae in crosses of *C. mwetiwel* females and *C. sp. 25* males.
Type locality: The type isolate was recovered from an unidentified substrate collected from the ground in the Pohnpei Botanical Garden in Nett on 13 December 2023. Overnight incubation in a Baermann funnel yielded thousands of worms. Five isofemale lines were established by picking single females on 15 December 2023. These five isolates (QG4797, QG5291, QG5306, QG5308, and QG5338) are all *C. mwetiwel.* Six additional isolates of this species were recovered from two other samples, both from an agroforest plot in U: a rotting sawa stem (taro, *Colocasia esculenta*) and a rotting banana flower.

bioRxiv preprint doi: https://doi.org/10.1101/2025.09.22.677770; this version posted September 24, 2025. The copyright holder for this preprint (which was not certified by peer review) is the author/funder, who has granted bioRxiv a license to display the preprint in perpetuity. It is made available under aCC-BY 4.0 International license.

Morphology notes: The male tail has the typical characteristics of Elegans supergroup species, including a heart-shaped fan with a serrated edge and terminal notch (Figure A1). Rays 1, 5, and 7 open dorsally. Ray 3 is equally distant from rays 2 and 4. Ray 4 is shorter and skinnier than ray 5. Spicules are long and slender with pointy tips, and the precloacal lip has the characteristic hook shape.
Reproduction: This species has separate males and females. Mating is in the parallel position on plates and females are oviparous.

Caenorhabditis nansapw **Rockman, Tintori, Nguyen, & Yomai** ***sp. nov.***
ZooBank identifier urn:lsid:zoobank.org:act:1693D8EC-B243-4B2B-A3EA-BF128572BF90
= *Caenorhabditis sp. 74* (temporary number)
Type material: The type isolate by present designation is isofemale culture QG4644, cryopreserved as a living stock at the *Caenorhabditis* Genetics Center, Minneapolis, MN. Derived from a single female collected in Kitti, Pohnpei, Federated States of Micronesia (coordinates N 6.8632, E 158.1765, elevation 253 m, 6 December 2023. The holotype is deposited at the *Caenorhabditis* Genetics Center.
Etymology: Nansapw is a Pohnpeian word for cultivated land, a reference to the type locality.
Diagnosis: This species reproduces with separate males and females. The species is diagnosed and delineated by fertile crosses with type isolate QG4644 in both directions, yielding fertile male and female offspring that are interfertile with one another and with both parental isolates in both directions. The species differs in its ITS2 DNA sequence from all other named species, including those listed in Félix *et al.* 2014; Huang *et al.* 2014; Ferrari *et al.* 2017; Slos *et al.* 2017; Kanzaki *et al.* 2018; Crombie *et al.* 2019; Stevens *et al.* 2019; Dayi *et al.* 2021; Sloat *et al.* 2022; Devi *et al.* 2025; and all other species described in this paper. The phylogeny inferred from transcriptome sequences places *C. nansapw* as sister to *C. ileile*. Reciprocal experimental crosses with *C. ileile* produced viable F_1s in both directions, but these F_1s did not produce any F_2s. Reciprocal crosses between each class of F_1 and each parental isolate produced zero or few embryos, which failed to hatch, in every case but one: F_1 females from the cross of *C. nansapw* females and *C. ileile* males, when crossed to *C. nansapw* males, produce a small number of offspring, some of which developed to adulthood. The clade of *C. nansapw* and *C. ileile* is sister to *C. imperialis,* with *C. mwetiwel* as the next outgroup. Reciprocal experimental crosses between *C. nansapw* and these other species produced infertile adults in all cases but one: crosses between *C. nansapw* females and *C. imperialis* males produced dead embryos only. Sister to this clade of four species is the clade of *C. sp. 25* and *C. sp. 49*. Reciprocal experimental crosses between *C. nansapw* and these species produced dead embryos, plus a small number of arrested L1 larvae in crosses of *C. nansapw* females and *C. sp. 25* males (Table S5).
Type locality: The type isolate was recovered from a rotting breadfruit collected from an agroforest plot at Pehleng, Kitti, on 6 December 2023. Overnight incubation in a Baermann funnel yielded approximately thousands of worms. Four isofemale lines were established by picking single females on 7 December 2023. These four isolates (QG4644, QG5287, QG5292, and QG5355) are all *C. nansapw*. Seventeen additional isolates of this species were recovered from five other samples: a rotting nihn fig (*Ficus tinctoria*) and two samples of rotting false durian in disturbed forest in Sokehs, an unidentified substrate at the Pohnpei Botanical Garden in Nett, and rotting breadfruit at an agroforest plot in U.
Morphology notes: The male tail has the typical characteristics of Elegans supergroup species, including a heart-shaped fan with a serrated edge and terminal notch (Figure A1). Rays 1, 5, and 7 open dorsally. Ray 3 is equally distant from rays 2 and 4. Ray 4 is shorter and skinnier than ray 5. Spicules are long and slender with pointy tips, and the precloacal lip has the characteristic hook shape.

bioRxiv preprint doi: https://doi.org/10.1101/2025.09.22.677770; this version posted September 24, 2025. The copyright holder for this preprint (which was not certified by peer review) is the author/funder, who has granted bioRxiv a license to display the preprint in perpetuity. It is made available under aCC-BY 4.0 International license.

Reproduction: This species has separate males and females. Mating is in the parallel position on plates and females are oviparous.

Caenorhabditis ileile* Rockman, Tintori, Nguyen, & Yomai *sp. nov.
ZooBank identifier urn:lsid:zoobank.org:act:61155085-3F94-4703-B045-ED9378A55FDC
= *Caenorhabditis sp. 75*
Type material: The type isolate by present designation is isofemale culture QG4848, cryopreserved as a living stock at the Caenorhabditis Genetics Center, Minneapolis, MN. Derived from a single female collected in Kitti, Pohnpei, Federated States of Micronesia (coordinates N 6.857742, E 158.2156, elevation 671 m, 8 December 2023. The holotype is deposited at the *Caenorhabditis* Genetics Center.
Etymology: Ileile is a Pohnpeian word meaning very high, a reference to the type locality.
Diagnosis: This species reproduces with separate males and females. The species is diagnosed and delineated by fertile crosses with type isolate QG4848 in both directions, yielding fertile male and female offspring that are interfertile with one another and with both parental isolates in both directions. The species differs in its ITS2 DNA sequence from all other named species, including those listed in Félix *et al.* 2014; Huang *et al.* 2014; Ferrari *et al.* 2017; Slos *et al.* 2017; Kanzaki *et al.* 2018; Crombie *et al.* 2019; Stevens *et al.* 2019; Dayi *et al.* 2021; Sloat *et al.* 2022; Devi *et al.* 2025; and all other species described in this paper. The phylogeny inferred from transcriptome sequences places *C. ileile* as sister to *C. nansapw*. Reciprocal experimental crosses with *C. nansapw* produced viable F_1s in both directions, but these F_1s did not produce any F_2s. Reciprocal crosses between each class of F_1 and each parental isolate produced zero or few embryos, which failed to hatch, in every case but one: F_1 females from the cross of *C. nansapw* females and *C. ileile* males, when crossed to *C. nansapw* males, produce a small number of offspring, some of which developed to adulthood. The clade of *C. nansapw* and *C. ileile* is sister to *C. imperialis,* with *C. mwetiwel* as the next outgroup. Reciprocal experimental crosses between *C. ileile* and *C. imperialis* produced dead embryos. Reciprocal experimental crosses between *C. ileile* and *C. mwetiwel* produced infertile F_1 adults. Sister to this clade of four species is the clade of *C. sp. 25* and *C. sp. 49*. Reciprocal experimental crosses between *C. ileile* and these species produced dead embryos, plus a small number of arrested L1 larvae in crosses of *C. ileile* females and *C. sp. 49* males (Table S5).
Type locality: The type isolate was recovered from rotting kotop (*Clinostigma ponapensis*) palm fruits collected from cloudforest along the ridge above Enipein, Kitti, on 8 December 2023. Overnight incubation in a Baermann funnel yielded many male and female *Caenorhabditis* larvae. Four isofemale lines were established by picking single females on 9 December 2023. One of these is QG4848, the type isolate of *C. ileile*. The other three isolates (QG4862, QG4729, and QG4884) are *C. pwilidak*. *C. ileile* is known only from this single isolate.
Morphology notes: The male tail has the typical characteristics of Elegans supergroup species, including a heart-shaped fan with a serrated edge and terminal notch (Figure A1). Rays 1, 5, and 7 open dorsally. Ray 3 is equally distant from rays 2 and 4. Ray 4 is shorter and skinnier than ray 5. Spicules are long and slender with pointy tips, and the precloacal lip has the characteristic hook shape.
Reproduction: This species has separate males and females. Mating is in the parallel position on plates and females are oviparous.

Caenorhabditis pwilidak* Rockman, Tintori, Nguyen, & Yomai *sp. nov.
ZooBank identifier urn:lsid:zoobank.org:act:D207B048-A366-4422-904B-A169AE0D5244
= *Caenorhabditis sp. 76*

bioRxiv preprint doi: https://doi.org/10.1101/2025.09.22.677770; this version posted September 24, 2025. The copyright holder for this preprint (which was not certified by peer review) is the author/funder, who has granted bioRxiv a license to display the preprint in perpetuity. It is made available under aCC-BY 4.0 International license.

Type material: The type isolate by present designation is isofemale culture QG4628, cryopreserved as a living stock at the *Caenorhabditis* Genetics Center, Minneapolis, MN. Derived from a single female collected in Kitti, Pohnpei, Federated States of Micronesia (coordinates N 6.906566, E 158.1817, elevation 541 m, 6 December 2023. The holotype is deposited at the *Caenorhabditis* Genetics Center.
Etymology: Pwilidak is the Pohnpeian word for native, as in reference to a native of Pohnpei.
Diagnosis: This species reproduces with separate males and females. The species is diagnosed and delineated by fertile crosses with type isolate QG4628 in both directions, yielding fertile male and female offspring that are interfertile with one another and with both parental isolates in both directions. The species differs in its ITS2 DNA sequence from all other named species, including those listed in Félix *et al.* 2014; Huang *et al.* 2014; Ferrari *et al.* 2017; Slos *et al.* 2017; Kanzaki *et al.* 2018; Crombie *et al.* 2019; Stevens *et al.* 2019; Dayi *et al.* 2021; Sloat *et al.* 2022; Devi *et al.* 2025; and all other species described in this paper. The phylogeny inferred from transcriptome sequences places *C. pwilidak* as distant sister to the clade of *C. kamaaina* and *C. oiwi*. Reciprocal experimental crosses between *C. pwilidak* and each of these species resulted in dead F_1 embryos in each case (Table S5).
Type locality: The type isolate was recovered from a rotting pwuhr (*Fagraea berteroana*) fruit collected from cloudforest along the ridge above Pehleng, Kitti, on 6 December 2023. Overnight incubation in a Baermann funnel yielded 20-30 *Caenorhabditis* larvae. Five isofemale lines were established by picking single females on 9 and 10 December 2023. All five isolates (QG4628, QG4643, QG5307, QG5325, and QG5332) are *C. pwilidak*. We collected an additional 801 isolates of *C. pwilidak*. These include 425 isolates from 71 substrates collected in three 1-m^2 grids of ninh (*Ficus tinctoria*) figs at Sokehs Ridge, and 381 collected from 101 samples representing 17 diverse substrate types in Kitti, Madolenhimw, Sokehs, and U.
Morphology notes: The male tail has the typical characteristics of Elegans supergroup species, including a heart-shaped fan with a serrated edge and terminal notch (Figure A1). Rays 1, 5, and 7 open dorsally. Ray 3 is equally distant from rays 2 and 4. Ray 4 is the same length and thickness as ray 5, which is different from its closest relatives *C. kamaaina* and *C. oiwi* (Crombie *et al.* 2019). Spicules are long and slender with pointy tips, and the precloacal lip has the characteristic hook shape.
Reproduction: This species has separate males and females. Mating is in the parallel position on plates and females are oviparous.

bioRxiv preprint doi: https://doi.org/10.1101/2025.09.22.677770; this version posted September 24, 2025. The copyright holder for this preprint (which was not certified by peer review) is the author/funder, who has granted bioRxiv a license to display the preprint in perpetuity. It is made available under aCC-BY 4.0 International license.

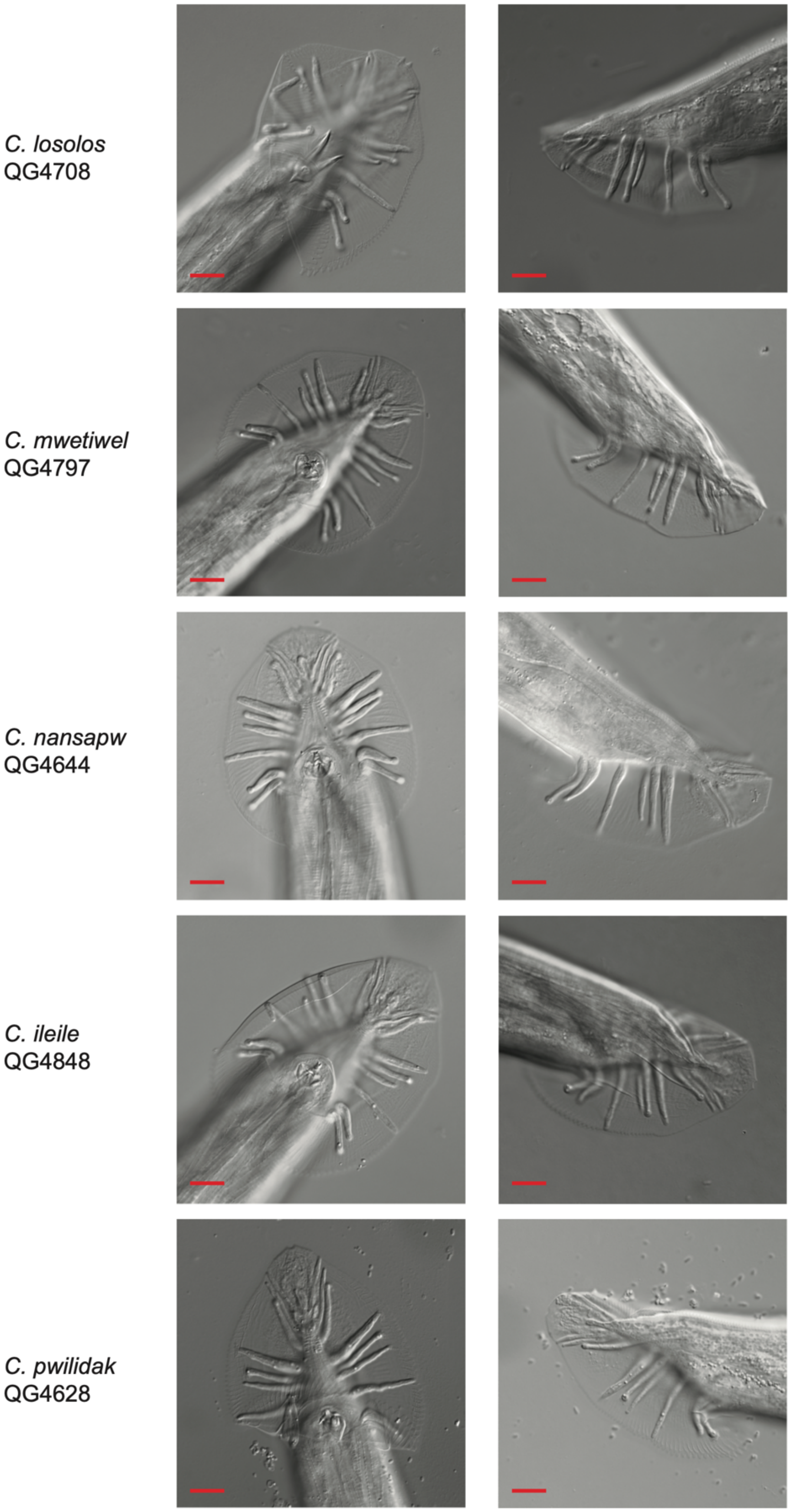

Figure A1. Ventral and lateral views of the tails of adult males, one day post-L4, for the type strains of each of five new species of *Caenorhabditis*. Scale bars are 10 μm.
